# Emerging Threats in Antifungal-Resistant Fungal Pathogens

**DOI:** 10.3389/fmed.2016.00011

**Published:** 2016-03-15

**Authors:** Dominique Sanglard

**Affiliations:** ^1^Institute of Microbiology, University Hospital Center, University of Lausanne, Lausanne, Switzerland

**Keywords:** antifungals, drug resistance, *Candida*, *Aspergillus*

## Abstract

The use of antifungal drugs in the therapy of fungal diseases can lead to the development of antifungal resistance. Resistance has been described for virtually all antifungal agents in diverse pathogens, including *Candida* and *Aspergillus* species. The majority of resistance mechanisms have also been elucidated at the molecular level in these pathogens. Drug resistance genes and genome mutations have been identified. Therapeutic choices are limited for the control of fungal diseases, and it is tempting to combine several drugs to achieve better therapeutic efficacy. In the recent years, several novel resistance patterns have been observed, including antifungal resistance originating from environmental sources in *Aspergillus fumigatus* and the emergence of simultaneous resistance to different antifungal classes (multidrug resistance) in different *Candida* species. This review will summarize these current trends.

## Introduction

Progresses in the therapy of human diseases have increased the survival of critically ill patients or patients with impaired function of their immune system. As a consequence, risk factors accumulate and favor the progression of other diseases, such as infectious diseases. Among these diseases, invasive fungal infections in humans represent a significant proportion. The most frequent fungal pathogens are *Candida*, *Aspergillus*, *Pneumocystis*, and *Cryptococcus* spp. It is estimated that these fungal species cause at least 1.4 million deaths worldwide per year (1). Compared to other microbial pathogens causing bloodstream infections, *Candida* spp. are ranked fourth among the most common agents of bloodstream infections, after other common bacterial pathogens (2). *Aspergillus* infections are the most common microbial infections in hematopoietic stem cell transplant (HSCT) recipients (3). About 30–50% of invasive aspergillosis patients still die, and the mortality from candidemia also remains high at ~50% (4).

There are only four major classes of antifungal drugs available to treat invasive fungal infections. They include polyenes, pyrimidine analogs, echinocandins, and triazoles (5). A fifth class (allylamines) is also existing; however, compounds of this class (for example, terbinafine) are used only for treating superficial dermatophytic infections (6). Polyenes, such as amphotericin B (AmB), have the ability to bind ergosterol and act as a sterol “sponge,” thus destabilizing membrane functions (7). Ergosterol is a major sterol of fungal membranes and is required for maintaining cell membrane integrity. AmB may exert intrinsic toxic effects in humans; however, this negative effect can be avoided by using liposome formulations (5). Pyrimidine analogs, such as 5-fluorocytosine (5-FC), are metabolized by fungal cells into fluorinated pyrimidines, which destabilize nucleic acids (RNA, DNA) and therefore result in growth arrest. 5-FC is used mainly for the treatment of *Cryptococcus* spp. meningitis and in combination with AmB (8). Echinocandins block the catalytic subunit of the β-1,3 glucan synthase and thus inhibit cell wall biosynthesis (9). In medical practice, triazoles are still the most used antifungals. These compounds target a specific step in the biosynthesis of ergosterol that catalyzes lanosterol 14α-demethylation (5). Fluconazole is the major triazole in clinical settings, probably due to its high oral availability and tolerability by patients.

## Antifungal Activity and Antifungal Resistance

The physical measure that determines antifungal activity is the reduction of growth *in vitro* as compared to drug-untreated cells. Antifungal activity is usually measured with standard dilutions in liquid media, although solid surface agars with drug gradients can be used as well (10). Two major protocols are currently used, both originating from major antifungal susceptibility testing subcommittees (CLSI, Clinical Laboratory Standards Institute; EUCAST, European Committee on Antimicrobial Susceptibility Testing). The protocols yield so-called minimum inhibition concentration (MIC) values (given in microgram per milliliter) as measures of antifungal activity. Although these protocols differ in several technical aspects, the agreement between the two methods in terms of antifungal activities is generally high (Table 1) (11). If a collection of isolates from the same species (for example, *Candida albicans*) is tested for susceptibility with a single agent (fluconazole), the resulting MICs will be distributed in a Gaussian bell-shaped manner. Such distribution helps to identify isolates (non-wild type isolates) differing from the general population of wild type isolates (12). The distinction between the two groups can be made with the help of the so-called epidemiological cut off (ECOFF) values. The ECOFF value is defined as the upper limit of the wild type population and in general will encompass about 95–99% of a given population for a specific agent. The ECOFF helps to detect non-wild type isolates that can typically be assigned as resistant isolates and may exhibit specific antifungal resistance mechanisms (13). *In vitro* resistance (or microbiological resistance) may be predictive of *in vivo* resistance (or clinical resistance). In order to achieve this, several studies have established clinical breakpoints (CBPs) for specific agents and specific fungal pathogens using several clinical parameters, including *in vivo* drug pharmacokinetics, resistance mechanisms, and clinical response. With MICs above CBPs, the success of therapy with a given agent is limited. For example, CBPs for fluconazole and *C. albicans* are declared as 2 and 4 μg/ml by EUCAST and CLSI, respectively. In a study in which candidemia episodes were enrolled (217) and treated with fluconazole monotherapy, infection-related mortality was significantly increased in *C. albicans* episodes with an MIC ≥2 μg/ml compared with those below this MIC target (20.6 versus 4.9%) (14). These results support well the proposed fluconazole CBPs of both EUCAST and CLSI. Table 1 gives an overview of current CBP of five important *Candida* spp. and currently available antifungal agents.

**Table 1:** **ECOFF and CBP of different antifungal agents and fungal species**.^a^

Species	Method	ECOFF (μg/ml)			CBP (μg/ml)					
		Fluconazole	Anidulafungin	Micafungin	Fluconazole		Anidulafungin		Micafungin	
					S^b^	R^b^	S	R	S	R
*C. albicans*	CLSI	0.5	≤0.12	≤0.03	2	4	0.25	0.5	0.25	0.5
	EUCAST	1	0.03	0.015	2	4	0.03	0.03	0.016	0.016
*C. glabrata*	CLSI	32	≤0.25	≤0.03	0.002	32	0.12	0.25	0.06	0.12
	EUCAST	32	0.06	0.03	0.002	32	0.06	0.06	0.03	0.03
*C. parapsilosis*	CLSI	2	≤4	≤4	2	4	2	4	2	4
	EUCAST	2	4	2	2	4	0.002	4	0.002	2
*C. tropicalis*	CLSI	2	≤0.12	≤0.12	2	4	0.25	0.5	0.25	0.5
	EUCAST	2	0.06	0.06	2	4	0.06	0.06	NA^c^	NA
*C. krusei*	CLSI	64	≤0.12	≤0.12	–^d^	–	0.25	0.5	0.25	0.5
	EUCAST	128	0.06	0.25	–	–	0.06	0.06	NA	NA

*^a^Data obtained from published studies (11, 13, 15–18)*.

*^b^Categorical discrimination between resistant (R) and susceptible (S)*.

*^c^NA, not available. EUCAST indicates that there is not yet available evidence that the species in question is a good target for therapy with the drug*.

*^d^“–” indicates that susceptibility testing is not recommended as the species is a poor target for therapy with the drug*.

*ECOFF, epidemiological cut-off; CBP, clinical breakpoint*.

A number of fungal species are not perturbed by specific antifungal agents at any concentrations. The absence of drug activity in a species that was not pre-exposed to the tested agent is also known as intrinsic resistance. Taking as example the response of *Candida* and *Aspergillus* spp. to fluconazole, it is known that wild type *C. albicans* is susceptible to fluconazole, whereas *Aspergillus fumigatus* and *C. krusei* are intrinsically resistant to this drug (19). It is reported that *Cryptococcus neoformans* is resistant to echinocandins, even if the target of these drugs, a β-1,3 glucan synthase, is *in vitro* highly sensitive to these drugs (20). It is thought that echinocandin resistance is rather due to the high content of other sugar polymers (β-1,6 glucans) in *Cr. neoformans*, since their biosynthesis is not affected by echinocandins (21). Nevertheless, antifungal resistance can be acquired *in vitro* by drug exposure or during therapy. Antifungal resistance can measured by elevated MICs as compared to those of a wild type population. Acquired antifungal resistance has been reported virtually for all existing antifungal agents and major fungal pathogens (22).

There are variable accounts on the frequency at which antifungal resistance occurs in hospitalized patients. The epidemiology of invasive fungal infections and associated resistance is based on data collected by sentinel and population-based surveillance programs. Here, we will review some data available for major fungal pathogens, including *C. albicans*, *C. glabrata*, *A. fumigatus*, and *Cr. neoformans*. Rates of resistance that are calculated from available data depend on the values that are used as CBP for given agents as was reported recently (23). The CBP recommended by the CLSI and EUCAST committees used to be very divergent for specific agents (for example, for fluconazole in *C. albicans*), but now tend to be more harmonized (Table 1). Given these divergences, it is sometimes difficult to make comparisons between old and more recent epidemiological studies (23). In any case, antifungal resistance rates in *C. albicans* are in general low. In a study from two different areas between 2008 and 2011, resistance to agents, such as fluconazole (CBP: ≥64 μg/ml) or echinocandins (CBP: ≥4 μg/ml), ranged between 1 and 2% in bloodstream isolates (24). Resistance rates in *C. glabrata* are higher than in *C. albicans*. According to data available in the ARTEMIS Antifungal Surveillance Program, *C. glabrata* increased as a cause of invasive candidiasis from 18% of all BSI isolates in 1992–2001 to 25% in 2001–2007. Fluconazole resistance rates in *C. glabrata* increased over the same period from 9 to 14% (CBP: ≥64 μg/ml) (25, 26). Resistance of *C. glabrata* to the class of echinocandins also reaches significant proportions. It was reported that, within a 10-year survey (2001–2010) in an US hospital (Duke University Hospital), echinocandin resistance rate increased from 4.9 to 12.3% (27). Similar trends are reported in Europe, although resistance rates range between 1 and 4% (28).

Antifungal resistance in *A. fumigatus* from clinical origin is mainly reported for the class of azoles, including itraconazole, voriconazole, and posaconazole. Rates of resistance are geographically variable. In general, resistance occurs when MIC values are above the ECOFF. These values are 1 μg/ml for itraconazole and voriconazole and 0.25 μg/ml for posaconazole (9). For example, resistance reached levels varying between 6 and 27% in the UK between 1997 and 2009, while it is up to 8% and only 0.6% in the Netherlands and USA, respectively (29).

*Cryptococcus neoformans* antifungal resistance is known for fluconazole (MIC ≥ 16 μg/ml). Fluconazole resistance has been described mostly in AIDS patients suffering cryptococcal meningitis. Although resistance rates were up to 28% in the early 90s (1990–1994), which corresponds to a period before the introduction of highly active anti-retroviral therapy (HAART), the current trends are around 1% (30, 31).

## Mechanisms of Antifungal Resistance

**Figure 1:**
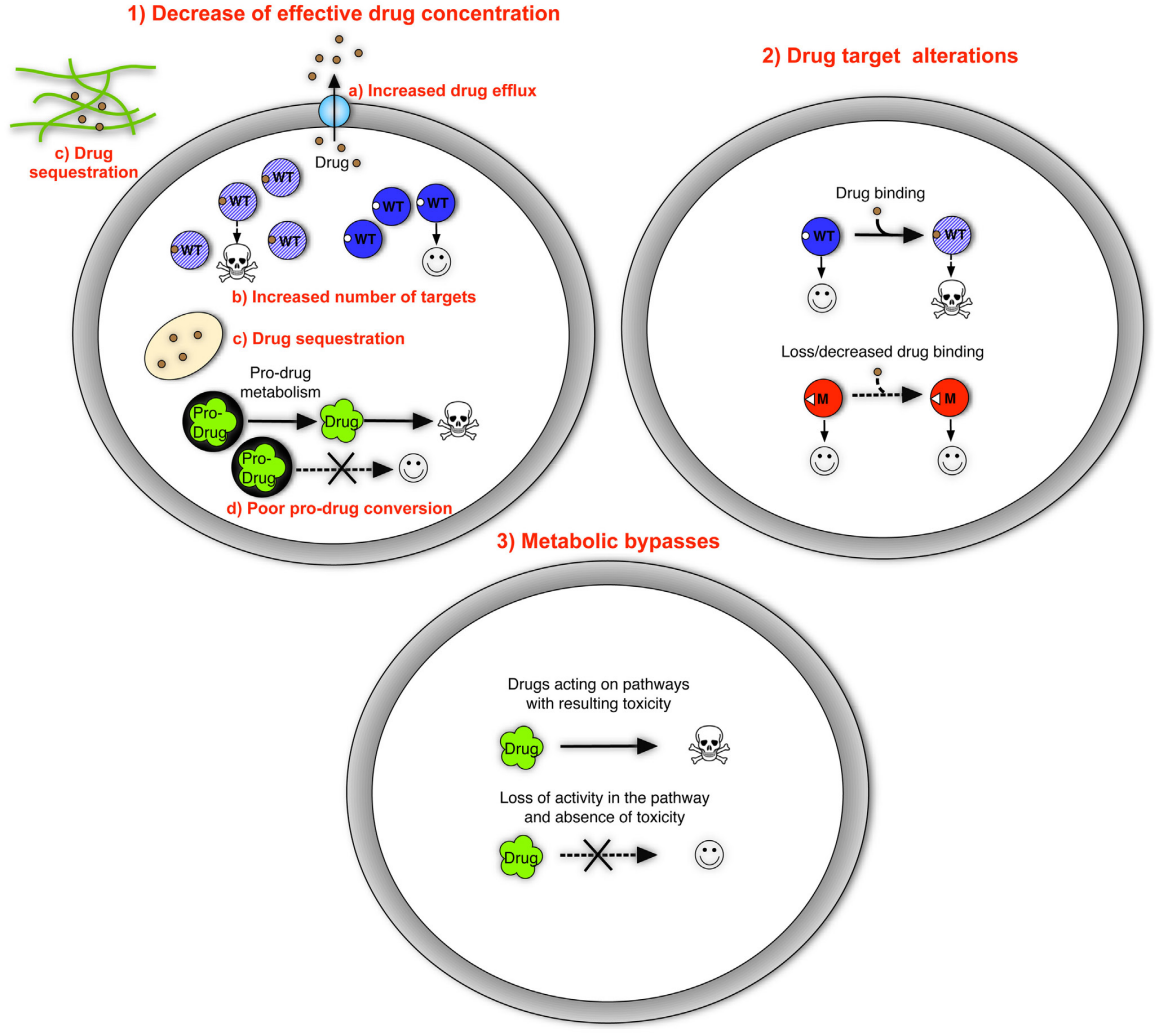
**The three basic resistance mechanisms to antifungal drugs**. They include (as listed in the text) (1) decrease of effective drug concentration with specific mechanisms including increased drug efflux, increased number of targets, drug sequestration of extracellular and intracellular origins, and poor pro-drug conversion; (2) drug target alterations; and (3) metabolic bypasses. Genome mutations are generally responsible for these three basic principles. Drug sequestration can be mediated by the formation of matrix polymers in biofilms, a state of cells that is not dependent on the occurrence of genome mutations. Wild type proteins are represented by blue circles catalyzing cellular functions; blue-shaded circles represent proteins blocked by drugs in which cellular functions are blocked causing decreased growth or death. Mutant proteins are represented by red circles. Drugs are represented with different symbols. Symbols: WT, wild type; M, mutant.

Mechanisms of antifungal resistance have been resolved at the molecular level for most antifungal agents and fungal pathogens. In principle, these mechanisms fall into distinct categories, including (1) decrease of effective drug concentration, (2) drug target alterations, and (3) metabolic bypasses. The major features for each of these principles are summarized below (Figure 1).

(1) Decreased effective drug concentrations can be achieved itself by several distinct mechanisms:
  (a) The drug intracellular concentrations can be decreased by active efflux.
    It is known that drug resistance can be mediated by the activity of several efflux transport systems, including ATP-binding cassette (ABC) transporters and transporters of the major facilitator superfamily (MFS). The analysis of fungal pathogen genomes has identified varying numbers of ABC transporters and MFS transporters with different topologies. *C. albicans* is predicted to contain 28 ABC proteins and 96 potential MFS transporters (32–34), whereas *C. glabrata* has at least 18 ABC transporters (35) and 33 MFS transporters (deduced from http://www.ebi.ac.uk/interpro/entry/IPR011701/taxonomy). Larger numbers of ABC and MFS proteins are found in *A. fumigatus* (45 and 275, respectively) and *Cr. neoformans* (29 and 192, respectively) (36, 37) (data available at http://www.membranetransport.org). ABC transporters are arranged in different subfamilies; however, they all contain membrane spanning domains and use ATP hydrolysis for drug transport. MFS transporters are transmembrane proteins, which use the electrochemical proton-motive force to mediate drug efflux. MFS are involved in multidrug resistance (MDR) (MFS–MDR transporters) function as proton antiporters and are classified into two groups: the drug:H+ antiporter-1 DHA1 family and the drug:H+ antiporter-2 DHA2 family (32, 38).
    Fungal ABC transporters have been arranged into several classes; however, only ABC transporters of the pleiotropic drug resistance (PDR) class are relevant for antifungal drug resistance. In *C. albicans*, the PDR class comprises the major transporters involved in azole resistance, including *CDR1* (for *Candida* drug resistance) and *CDR2*, and also other transporters not shown yet to be involved in antifungal resistance (*CDR3*, *CDR4*, *CDR11*, and *SNQ2*). Basically, the upregulation of both *CDR1* and *CDR2* mediates azole resistance by enhanced drug efflux and reduces azole accumulation in some *C. albicans* clinical strains (39). Several other ABC transporters known to be involved in azole resistance by their upregulation are the *C. glabrata CgCDR1*, *CgCDR2*, *CgSNQ2* genes, and *AFR1* from *Cr. neoformans* (40). In *A. fumigatus*, the association between azole resistance and transporter upregulation is less clear. The ABC transporter *atrF* was shown as upregulated in an azole-resistant clinical isolate; however, this could not be firmly attributed as a cause of resistance (41). *AfuMDR4* was strongly upregulated in several itraconazole-resistant laboratory-derived mutants (42). Transcriptional profiling revealed transporter genes whose expression was induced in response to voriconazole (43). These included five ABC transporter genes (designated *abcA–E*) and three MFS transporter genes (designated *mfsA–C*). The *abcA* gene, renamed *cdr1B*, is the only known transporter gene with a direct role in azole resistance in *A. fumigatus* (44). *AfuMDR3*, a MFS transporter in *A. fumigatus*, was found as upregulated in a collection of itraconazole-resistant laboratory-derived mutants; however, its participation in azole resistance of clinical isolates remains elusive (42). MFS involved in the development of azole resistance in clinical isolates are restricted to *MDR1* from *C. albicans* and *C. dubliniensis*. *MDR1* is upregulated in specific strains, which results in enhanced azole efflux (45, 46). *FLU1* (for Fluconazole resistance) from *C. albicans* is another MFS, and heterologous expression in *S. cerevisiae* revealed that it served as a fluconazole efflux transporter (47). Until now, however, no studies have shown the participation of *FLU1* in azole resistance in clinical isolates.
    Upregulation of ABC and MFS transporters is mediated by specific regulators in resistant fungal pathogens. In *C. albicans*, *CDR1* and *CDR2* are known to be regulated by a zinc cluster finger transcriptional regulator called *TAC1* and *MDR1* by another regulator called *MRR1* (48, 49). Mutations (gain-of-function or GOF mutations) in these regulators have been described, and they confer an hyperactivation state that does not require additional stimulation, thus explaining the inherent high expression levels of the transporters in drug-resistant isolates (50, 51). Other transcriptional regulators of drug transporters relevant to azole resistance, such as *PDR1*, have been described in *C. glabrata* (52, 53).
  (b) The drug target is overexpressed.
    By increasing the number of drug targets, the effective drug concentration needs to be also increased to saturate all target molecules, which results in drug resistance. For example, *ERG11* upregulation has been associated with azole resistance in *C. albicans*. This transcriptional regulation is mediated by a zinc cluster finger transcription factor called *UPC2*. As in the case of other drug resistance transcriptional regulators, GOF mutations in *UPC2* have been described and result in upregulation of various genes, among which is *ERG11* (54). Upregulation of *Cyp51A* is also known in azole-resistant *A. fumigatus* isolates; however, the upregulation is mediated by duplication of 34- and 42-bp elements (*trans*-regulation) in the *Cyp51A* promoter. This duplication is associated with specific Cyp51A mutations (L98H, Y121F/T289A) (55). These combined mutation signatures are enriched in azole-resistant *A. fumigatus* isolates originating from the environment that probably arose from the use of azoles in the agriculture (29).
  (c) The drug is sequestered in extra- or intracellular compartments.
    Fungal pathogens have the ability to sequester drugs within extracellular compartments. Several fungal pathogens, including *Candida* and *Aspergillus* spp., are able to form biofilms in specific growth conditions (56). Biofilms are multicellular structures in which cells form a dense network that is covered by the so-called matrix. The matrix is composed of different elements in *C. albicans* biofilms, including several cell wall polymers (57). Biofilm formation is known to be associated with resistance to several drugs, including azoles, polyenes, and pyrimidine analogs (58). Interestingly, recent data showed that the matrix participates to this process by its capacity to sequester antifungal agents. This process has been clearly documented for fluconazole (57, 59) and was suggested for AmB in *C. albicans* (60).
    Much less is known in drug sequestration in intracellular compartments. A single report document the accumulation in *C. albicans* of fluconazole in organelles that were described as vesicular vacuoles. Whether or not this type of mechanism could occur in other isolates remains unknown (61).
  (d) A pro-drug is poorly converted to an active drug.
    Poor drug metabolization as a principle of antifungal resistance is also observed when 5-FC resistance occurs. 5-FC is a pro-drug, which is metabolized by cells into fluorinated pyrimidine analogs, thus inhibiting nucleic acid and protein biosynthesis. After import into cells, cytosine deaminase converts 5-FC into 5-fluorouridine (5-FU) and therefore the deficiency of this step decreases further processing and toxicity of the drug. Mutations in cytosine deaminase in *C. albicans* (*FCA1*) (62) and *C. glabrata* (*FCY1*) have been reported, resulting in 5FC resistance (63, 64).
(2) Drug target alterations have been reported for at least two classes of antifungal agents, including azoles and echinocandins. The targets of these two drugs are a 14α-lanosterol demethylase and a β-1,3 glucan synthase, respectively. Lanosterol demethylase is encoded by *ERG11* in *C. albicans* and *Cyp51A* and *Cyp51B* in *A. fumigatus*. Mutations in *ERG11* resulting in non-synonymous amino acid substitutions that are present in azole-resistant *C. albicans* isolates are numerous and were shown to decrease the affinity of the target to azoles (65). The effects of *ERG11* mutations have different outcomes on azole MICs that depend on structural features of azole drugs. Although most known mutations decrease affinity to fluconazole, they have only a moderate effect on posaconazole affinity (66). In many cases, simultaneous *ERG11* mutations can be present on the same *ERG11* allele and be accompanied by drug transport modifications, thus resulting in azole-resistant isolates with high MIC values against azoles (for example, fluconazole MIC > 128 μg/ml) (67). Mutations in lanosterol demethylase genes from azole-resistant *A. fumigatus* isolates have been only reported in *Cyp51A* until now. Single *Cyp51A* mutations are sufficient to confer high level resistance to azoles in this species (68). As in the case of *ERG11*, *Cyp51A* mutations have different impact on MICs that depend on the azole structure (69).
  Decreased affinity to the target is also known for echinocandins. β-1,3 glucan synthases are encoded by *FKS* genes in different fungal species. Up to now, echinocandin resistance has been attributed to specific mutations leading to amino acid substitutions in two different regions of these genes (Hot spot 1 and 2 or HS1 and HS2). *FKS1* mutations have been reported in these two regions (HS1: region 640–650 and HS2: 1345–1365) in clinical isolates of *C. albicans* (70). Equivalent mutations in the HS1 of *FKS2* (an homolog to *FKS1*) of *C. glabrata* and *FKS1* of *Candida lusitaniae* (71, 72), *C*. *tropicalis*, and *C. krusei* (73) have been reported. Some *Candida* species (the *Candida parapsilosis* family, including *C. parapsilosis*, *C. orthopsilosis*, and *C. metapsilosis*) exhibit intrinsic low susceptibilities to echinocandins. *FKS1* genes in these species contain a natural polymorphism (P660A at the 3′-extremity of HS1) enabling decreased affinity of the β-1,3 glucan synthase to echinocandins. However, this natural *FKS1* polymorphism of these species has less impact than those acquired by mutations, since these *Candida* species still respond to echinocandin therapy (74).
(3) Metabolic bypasses occur when given metabolic pathways are perturbed by loss or strong decrease of specific functions. Metabolic bypass can be compared to compensatory mechanisms in which cells divert the toxic effects exerted by some antifungal agents. For example, resistance to azoles can be mediated by loss-of-function mutations in the gene *ERG3* that encodes a sterol Δ^5,6^ desaturase. If active, the gene product converts 14α-methylated sterols that arise from azole exposure into a toxic 3,6-diol derivative (75). Fungi unable to produce this metabolite acquire azole resistance. Several studies have reported *ERG3* loss-of-function mutations to account for azole resistance (76–79). Due to a deficiency in ergosterol biosynthesis, these isolates can be, however, less competitive than wild type isolates in conditions encountered in the host. As a result of loss-of-function of *ERG3* in specific mutants, ergosterol is absent from cell membranes. This way, the mutants escape the toxic effect of AmB, which normally acts as a “sponge” for ergosterol to rapidly destabilize membrane functions (7). Several other mutations in the ergosterol biosynthesis pathway (*ERG6*, *ERG24*, and *ERG2*) lead to the same effect and have also a compensatory effect (80–82).
  A mutation in the gene *FUR1* encoding uracil phosphoribosyltransferase decreases the conversion of 5-FU, which is produced from 5-FC deamination (see above), into a toxic metabolite (5-FC monophosphate). Thus, the toxic effect of 5-FC cannot be exerted (83).

## Antifungal Resistance from Environmental Origin

Azole antifungal agents are not only widely used in medicine but they also largely contribute to crop protection in agriculture and are used to preserve materials from fungal decay (84). Therefore, *A. fumigatus*, as a ubiquitous fungus, is likely to come into contact in the environment with the same substance class that is used in medicine. A first report on azole resistance from environmental isolates was published in 2007 from the Netherlands (85). In this study, the authors were able to identify a mutation in the azole target *Cyp51A* (a L98H substitution), which was associated with a 34-bp tandem repeat (TR34) in the gene promoter. Interestingly, the same mutation was recovered from patient samples, strongly suggesting that the *Cyp51A* L98H/TR34 mutation was acquired from environmental isolates. This mutation results in resistance to all medical azoles (pan-azole resistance). One argument that is crucial to support environmental acquisition of azole resistance is that between 64 and 71% of patients with IA due to an azole-resistant *A. fumigatus* isolate had never received azole treatment before (86). There are concerns that azole resistance could become a global public health threat, since fungal spores can disperse easily by circulating air flows across long distances (84). Currently, environmental resistance is documented in several other European and Asian countries and America (29, 87, 88). Other *Cyp51A* mutations than the L98H/TR34 are now also reported from environmental isolates, including TR46/Y121F/T289A (89), as well as others (G54A and M220I) that were until now exclusively recovered from clinical isolates (90). These data suggest that systematic surveillance programs should be initiated worldwide. The use of azoles in the environment will be difficult to restrict, unless scientists raise better public and political awareness on this problem.

## Multidrug Resistance: A Pattern of Concern

Antifungal resistance has been observed in most occasions as a process involving resistance to single classes of drugs. Within the same class, several different agents can exist. Examples are for the classes of azoles (fluconazole, itraconazole, voriconazole, posaconazole, and isavuconazole) and echinocandins (caspofungin, micafungin, and anidulafungin).

Specific resistance mechanisms can result in cross-resistance to several drugs of the same class. It is known that the expression of ABC transporters (i.e., *CDR1* or *CgCDR1*) mediate cross-resistance to all azoles used in medicine (66). Likewise, specific *FKS1* mutations in *C. albicans* (F641S, S645Y) yield cross-resistance to all echinocandins (91).

Multidrug resistance is the simultaneous resistance to at least two different classes of antifungal agents. In the recent years, reports documenting cases of MDR in fungal pathogens have been published. We will give here an overview of the latest trends in the emergence of MDR.

## MDR Between Azoles and Amphotericin B

Many fungal infections are treated with different antifungal agent classes, including azoles and polyenes. MDR between these two classes could be mediated expectedly by different genomic mutations; however, it has been reported that it is sufficient to harbor only loss-of-function mutations in *ERG3* to result in simultaneous MDR against azoles and AmB. Such mutations have been reported in *C. albicans* (79, 92–94) and *C. dubliniensis* (95). Other *ERG* gene defects may also confer MDR to both drug classes, such as the loss-of-function mutation in *ERG2* observed in *C. albicans* (82).

Nevertheless, some specific isolates may show simultaneous mutations in several genes as a cause of MDR. This was reported in *C. tropicalis* by *ERG3*/*ERG11* loss-of-function mutations (77, 82) and in *C. albicans* by *ERG11*/*ERG5* mutations (92).

## MDR Between Azoles and Echinocandins

Echinocandins are being increasingly used for the therapy of fungal infections, especially those caused by *Candida* spp. Resistance to echinocandins logically appeared soon after its introduction in medicine in 2005 (96). A first report of MDR to caspofungin and azoles in *C. glabrata* isolated from blood cultures was made in 2010 after caspofungin therapy (97). Resistance mechanisms were combining mutations in the β-1,3 glucan synthase *FKS2* (S663P) and ABC transporters upregulation. Closely related isolates became resistant to 5-FC after therapy with this drug; however, it was still susceptible to the two other drugs. These isolates exhibited a non-synonymous mutation (G190D) in *FUR1*, which probably accounted to decrease 5-FC toxicity.

The current trends show that the highest proportion of resistant isolates is from the species *C. glabrata* (70). Other observations were made recently on MDR with both azoles and echinocandins in *C. glabrata*. Among echinocandin-resistant isolates sampled between 2008 and 2013 in two US surveillance hospital sites, 36% were also resistant to azoles (98). Similar observations were reported in another US site between 2005 and 2013, in which 10.3% *C. glabrata* isolates from cancer patients were resistant to caspofungin and from which about 60% had a MDR phenotype with azoles (99). The data of this study also indicated that caspofungin exposure alone could induce MDR without azole exposure. Here, resistance mechanisms were not systematically investigated in these isolates; however, they are likely to involve *FKS1*/*FKS2* and *PDR1* mutations. This MDR pattern is therefore of concern, especially when considering that very few therapeutic alternatives are available.

## MDR Beyond Two Drug Classes

Combining resistance for more than two drug classes is not a frequent observation in clinical isolates. However, a few cases have emerged recently and highlight the capacity of specific pathogens to adapt to strong antifungal selective pressure.

A recent case illustrated the evolution of MDR in *C. albicans* sequential isolates taken from a patient at different sites (oropharynx, esophagus, feces, and colon) treated over time (100). The isolates were related to each other as confirmed by genotyping methods. The evolution of drug resistance followed the course of drug treatments. Fluconazole treatment induced first a GOF mutation in *TAC1* with corresponding azole resistance (MIC fluconazole >16 μg/ml). Caspo- and anidulafungin treatment resulted in resistance (MIC caspofungin >32 μg/ml) with a corresponding *FKS1* mutation (S645P). Lastly, AmB treatment established AmB resistance (MIC > 32 μg/ml) with a loss-of-function mutation in *ERG2*. All three mutations were conserved in the final MDR strain (100). MDR evolution took place within a time lapse of 5 years.

Another example originates from a *C. lusitaniae* infection in a young immunocompromised patient with severe enterocolitis and visceral adenoviral disease (71). *C. lusitaniae* isolates were recovered from blood cultures and stools over a period of 3 months. Very early at onset of therapy, fully susceptible isolates were recovered. Caspofungin regimen resulted in detection of resistance (MIC = 4 μg/ml) with a corresponding *FKS1* mutation in HS1 (S638Y). This resistance coincided with AMB resistance, although not administered simultaneously. The therapy was continued by fluconazole from which resistance rapidly emerged (MIC = 32 μg/ml). Fluconazole resistance could be associated with upregulation of a MFS transporter (*MFS7*) but was also accompanied by 5-FC resistance, even if no 5-FC was administered. Combination therapy with caspofungin and voriconazole was next attempted, and isolates with simultaneous resistance to caspofungin, fluconazole, and 5-FC were detected. These isolates exhibited another *FKS1* mutation (S631Y) and *MFS7* upregulation. This study highlighted a very dynamic property of *C. lusitaniae*, which responded quickly to antifungal exposure. In this specific type of abdominal disease, it is believed that a reservoir of fungal cells was present with mixed MDR genotypes, and, depending on the drug treatment regimen, dominant populations could emerge (71).

## Conclusion and Perspectives

A consequence of the use of antifungal agents in the therapy of fungal diseases is to face antifungal resistance in fungi. The extent of the problem is variable and depends on the type of fungus, the type of antifungal agent and on the geographical location of hospitals. In the recent years, however, reports on novel resistance profiles have appeared, and one of the most problematic is the development of MDR. It seems that, up to now, MDR occurs mostly in the species *C. glabrata*, especially since the introduction of echinocandins in the clinic. The reasons behind MDR in this pathogen are still unclear. Since *C. glabrata* harbors a haploid genome, single genetic events are sufficient to express phenotypes, which are less the case for diploid organisms (for example, *C. albicans*). One other reason is that the genome context of *C. glabrata* may facilitate the occurrence of genetic events. Very recent data from the laboratory of D. Perlin suggest that some *C. glabrata* exhibit much higher mutations rates than others (hypermutator phenotype) (101). With the appearance of these novel resistance profiles, alternative therapeutic approaches are required and novel antifungal agents need to be identified.

## Author Contributions

The author confirms being the sole contributor of this work and approved it for publication.

## Conflict of Interest Statement

The author declares that the research was conducted in the absence of any commercial or financial relationships that could be construed as a potential conflict of interest.
